# Sharing whilst caring: solidarity and public trust in a data-driven healthcare system

**DOI:** 10.1186/s12910-020-00553-8

**Published:** 2020-11-03

**Authors:** Ruth Horn, Angeliki Kerasidou

**Affiliations:** The Ethox Centre and Wellcome Centre for Ethics and Humanities, University of Oxford, Old Road Campus, Oxford, OX3 7LF UK

**Keywords:** Solidarity, Solidaristic healthcare system, Public trust, Data sharing, Private companies, Data-driven technologies

## Abstract

**Background:**

In the UK, the solidaristic character of the NHS makes it one of the most trusted public institutions. In recent years, the introduction of data-driven technologies in healthcare has opened up the space for collaborations with private digital companies seeking access to patient data. However, these collaborations appear to challenge the public’s trust in the.

**Main text:**

In this paper we explore how the opening of the healthcare sector to private digital companies challenges the existing social contract and the NHS’s solidaristic character, and impacts on public trust. We start by critically discussing different examples of partnerships between the NHS and private companies that collect and use data. We then analyse the relationship between trust and solidarity, and investigate how this relationship changes in the context of digital companies entering the healthcare system. Finally, we show ways for the NHS to maintain public trust by putting in place a solidarity grounded partnership model with companies seeking to access patient data. Such a model would need to serve collective interests through, for example, securing preferential access to goods and services, providing health benefits, and monitoring data access.

**Conclusion:**

A solidarity grounded partnership model will help establish a social contract or licence that responds to the public’s expectations and to principles of a

solidaristic healthcare system.

## Background

The National Health Services (NHS) UK is a solidarity-based institution, founded on the principles that: (1) all should contribute to it according to their means (through taxation); (2) all should be offered best care according to their needs; and (3) services should be free for all at the point of access. These foundational principles have made the NHS one of the most trusted institutions in the UK [1]. In recent decades, and particularly since the 1990s, private companies have been entering the care provision domain in the UK. Lately, partnerships between the public healthcare provider and private companies have intensified. Reasons for this closer collaboration between the two sectors is the increasing reliance of medicine and healthcare on new data-driven technologies, such as genomics medicine and artificial intelligence, but also the recognition of the financial value of NHS data to the international digital market [2]. With an ageing population and after years of austerity, the NHS is struggling to meet the care needs of the population. Data-based technological solutions, such as artificial intelligence applications, have been proposed as a way of effectively responding to the funding and staffing crisis faced by the NHS [3], which has opened the door to private digital companies developing such tools. The government has realised that the NHS, as the single largest integrated health care provider in the world, is in a unique position to attract ‘investments across the health tech and pharmaceutical sector’ [4]. Since 2018, the two governmental industrial strategy deals centred on data-driven technologies to create and grow new industries in the UK, and on collaborations between the life sciences sector and industry, universities and charities to demonstrate the value of the NHS as a partner in this new industrial vision [4].

The use of NHS data to drive economic growth rather than solely benefiting patients, is challenging the solidaristic character of the healthcare system. In 2018, the Secretary of State for Health and Social Care pleaded for a more tech- and big data-driven NHS, but also acknowledged the importance of building and maintaining public trust in the healthcare system [5]. And yet, there is increasing concerns regarding the use of patient data, and the motivations of private companies partnering with the NHS that appear contrary to public interest [6]. In light of the changes introduced by the use of data sciences in healthcare, some scholars have called for the need of a new ‘social contract’ [7] or ‘social licence’ [8] between the healthcare system, patients and society as a whole.

In this paper we explore how the opening of the healthcare sector to private digital companies challenge the existing social contract and the NHS’s solidaristic character, and how this impacts on public trust. We start by critically discussing different examples of partnerships between the NHS and private companies that collect and use data. We then analyse the relationship between trust and solidarity, and investigate how this relationship changes in the context of private companies entering the healthcare system. Finally, we propose ways for the NHS to maintain its solidaristic character and public trust by putting in place solidarity grounded partnerships with companies seeking to access patient data.

## Methodology

This paper is a theoretical analysis arguing for the adoption of solidarity grounded partnerships to govern data sharing between public healthcare systems and commercial or private companies accessing patient data. Our analysis focuses on the UK context and draws on bioethics literature, surveys on public trust in health care and/or data sharing, prominent case studies that gave rise to relevant debates in the UK, and official reports.

## Discussion

### NHS data mining and public trust

The delivery of a health service in the twenty-first century increasingly relies on data. A number of data-driven technologies and applications are entering the space of healthcare, including genomics medicine and artificial intelligence (AI), promising more accurate prediction and prevention of disease, improved health outcomes and more efficient use of resources [9]. A vast array of clinical, genomic, demographic, environmental and social data are needed in order to fulfil these promises aiming to improve the performance and running of healthcare systems. Data has always been important for healthcare improvement. For a long time, however, only certain types of data, for example clinical, health records and clinical trial data, were considered to have direct health relevance. In recent years, and with the advancement in big data science, a number of seemingly irrelevant and unrelated information have become important, as they can provide valuable insights into the health and health-related behaviour of individuals and populations. For example, people’s online behaviour could provide important information about the spread of infectious diseases such as flu [10]. This growing utility and relevance of different types of data for health, has opened up the field of healthcare to types of companies and institutions that were not part of the healthcare landscape before, and created the conditions for collaboration between the public healthcare sector and commercial entities, such as digital companies (e.g. Google) and online retailers (e.g. Amazon).

One of the major holders of health-related data in the UK is the NHS. As the main healthcare provider, the NHS keeps a vast number of different types of data, from general practitioners’ notes, to clinical test results, to genetic and genomic information. These data, protected by the principle of confidentiality, traditionally stayed within the boundaries of the healthcare system [11, 12]. Identifiable patient information has only been used for the direct benefit of the patients. Anonymised patient data have been used by the NHS to improve care provision too; for example, hospital episode statistics have been collected and analysed by the NHS since 1989 [13]. Yet, in recent years, with the recognition of the health but also economic value NHS data hold, and with the expansion of the healthcare space to non-traditional stakeholders (e.g. digital companies), new projects and practices regarding data collection, use and sharing are emerging.

In 2013, the UK government tried to expand the NHS data sharing practice, by introducing a new scheme called care.data [14]. This new scheme aimed to bring together health and social care data from across the NHS, and share them, under strict conditions protecting patient privacy, with academic and commercial researchers to develop new treatments and improve the performance of the healthcare system. Some of the aims of care.data were to support patient choice, promote transparency, improve outcomes for patients, and also drive economic growth [8]. Within a few months of the announcement of the new scheme, however, more than a million of patients opted-out of the care.data system, and by 2016, the scheme was discontinued [15]. The main reasons that led to the collapse of the care.data scheme was the lack of communication of the government’s plans and intentions to the general public, and the people’s concern that NHS data will be shared with private commercial companies. In 2016, the Wellcome Trust published a report on a study regarding people’s attitudes to health data sharing with commercial actors. The study found that whereas the vast majority of the population was willing to share their data within the NHS and for non-commercial research, for many, sharing data with commercial companies solely for private benefit and without clear public benefit constituted a ‘red line’ that should not be crossed [16]. In their analysis of the care.data case, Carter et al. [8] observe that the scheme ‘failed to adequately secure a social licence’ i.e. society’s agreement, because of: (1) defects in the warrants of trust provided for care.data, (2) the implied rupture in the traditional role, expectations and duties of general practitioners, and (3) uncertainty about the status of care.data as a public good’. Such a social licence, the authors argue, is needed when a public and private body’s activities go ‘‘beyond compliance’ with legal requirements’ [8].

Around the same time of the care.data scheme, the UK Government announced another initiative aiming at collecting patient data to support individualised care, medical innovation and economic development. In 2013, Genomics England, a company owned by the UK Department of Health, was set up to oversee the 100,000 Genomes Project, a UK Government initiative [17]. The aim of this initiative was to sequence 100,000 genomes, and to use the data generated for medical and scientific research. The original focus of the project was on rare diseases, infectious diseases, and some types of cancer. After meeting its primary target of 100,000 genomes, in 2018, the project expanded its remit, aiming to sequence five million genomes over the following five years. As the success of the project heavily relied on patients’ willingness to donate their data, the Chief Executive of Genomics England acknowledged that ‘building trust in this exciting and revolutionary area of medical science is absolutely essential to its success’ [18]. The project set up an independent ethics committee and endeavoured right from the beginning for greater transparency, public involvement and also technological solutions to address data security concerns as a way of ensuring public trust. In 2019, Genomics England published a report on public views on the use and sharing of genomic data [19]. Whilst survey participants expressed their support of the overall initiative, once again, one of their main concerns was the involvement of private companies and the sharing of data with private institutions that did not seem to share the solidaristic ethos of the NHS. Participants seemed to trust the NHS to use their data for the common good, but their trust did not extend to include other actors that might be given access to their information. The report recommended that public trust can only be built and maintained if the NHS keeps embodying its core values of reciprocity, altruism, and solidarity.

Despite the failure of care.data, and people’s concerns about the increasing access private companies are given to patient information, the government continued with its plans to extract value from NHS data. In 2016, the NHS agreed a deal with DeepMind, an AI company, to access sensitive data of more than one million patients. DeepMind was later acquired by Google, and in 2019, five NHS Trusts (legal entities providing hospital services, community services and/or other aspects of patient care) agreed data access contracts with Google Health [20]. In 2019, there was a similar deal with Amazon where the technological company was given free access to NHS patient data in order to develop the Amazon Alexa health device that can offer health advice to its customers [21]. Subsequently, the government that signed the contract has been heavily criticised, among others by privacy organisations, for lacking transparency and prioritising commercial interest [22].

The public’s concern with and apprehension regarding the sharing of their health data with bodies outside the NHS, and particularly with commercial entities is well documented [6, 16, 18]. However, the digital revolution that the UK Government is envisaging and promoting for cutting-edge healthcare depends on the public’s co-operation and continuing trust in the NHS and other relevant institutions [5]. Yet, in the emerging healthcare landscape that involves collaborators that do not seem to share the solidaristic core values of the NHS, maintaining public trust appears increasingly challenging.

### Solidarity and public trust in the healthcare system

Solidarity is the founding principle of publicly funded healthcare systems, and has occupied an important place in the development of healthcare policies in Europe, since the late nineteenth century [23]. In public healthcare systems, access to services and care is organised by the government and funded through either mandatory payments of an insurance premium (e.g. in Germany, France and Belgium) or taxation (e.g. in the UK and Canada). As ter Meulen argues, individuals accept state enforced solidarity for two reasons: on the one hand out of ‘self-interest’ and on the other hand out of ‘a feeling of responsibility […] togetherness and commitment to the common good’ [24]. It is this collective sense of social cohesion and interdependence what distinguishes publically funded from private healthcare systems [25].

Although solidarity has never been a widely used notion in the political and social discourse in the UK [26], the NHS’s founding principles of universality, equity, comprehensiveness, quality, free services, and public funding [27], have laid the ground for a healthcare system that distributes resources and free care based on need rather than ability to pay. In this way, the NHS as a public institution is demonstrating its commitment to the common good, and is promoting a sense of mutual responsibility, which are central to the concept of solidarity [28]. Solidarity has two meanings; one descriptive and one normative. First, and this goes back to Durkheim, solidarity describes the social bond that ties individuals together through acknowledgement of reciprocity and interdependence [29]. Second, this bond or feeling of social cohesion implies that members of a group have a set of normative expectations of what they owe to each other, that is how to behave in a solidaristic way [30]. What distinguishes solidarity from other related concepts such as justice or altruism, is its relational aspects that makes individuals identify with each other and ‘stand up for/with/as’ to promote joint interests [31].

The expectation of solidarity and the lived experience of it can promote public trust in social institutions. According to Uslaner, trusting societies are the ones that share resources equally amongst their members [32]. Similarly, Rothstein and Stolle argue that solidaristic welfare programmes that do not differentiate between individuals (e.g. selective welfare programmes) but are universal in coverage contribute to the development and establishment of trust between society members [33]. Thus, public trust is built on the reasonable belief that public institutions will serve the common good and represent collective interests [34]. This is supported by a collective feeling of commonality and mutuality reinforced by institutional structures and public policies.

One example of the relationship between public trust and solidarity is the NHS. Since its foundation, and despite a general decrease of trust in public institutions among Britons [1], the NHS has been, and still is, one of the most appreciated and respected public institutions in the UK [35, 36].Years of underfunding, which led to a decrease and underperformance of its services, have fuelled concerns amongst the population of whether the NHS can be maintained in its current form [37]. A survey of 2017 showed that a large majority of the population (around 90%) are concerned about the NHS maintaining its foundational principles such as that the NHS should be free at the point of delivery, provide a comprehensive service available to everyone, and be primarily funded through taxation [37, 38]. More than half of the survey respondents indicated that they are willing to support the NHS and its ability to provide good quality care for all through solidaristic measures such as increased taxation [38]. This shows that it is not only the reliance on the NHS’s skills and ability to provide good care that drives public trust, but also its solidaristic character as an indication of the NHS’s moral attitude of good will towards those in need of care [39].

Despite the wide support among the population for a healthcare system that is committed to the common good and promotes mutual responsibility, for example, through taxation, the UK governments since 2010 have sought to resolve the NHS funding problem mainly through partnerships with commercial entities such as those seeking access to patient data. These partnerships with profit-oriented collaborators appear to contradict the solidaristic character of the NHS that is based on accepting mutual responsibility, recognising the importance of the community, and on developing feelings of togetherness. People’s concern and discontent regarding these developments are expressed in the form of mistrust towards the government and its new profit-driven partners. As described above, these partnerships often lack transparency and a ‘social licence’ to operate outside of the ‘societal seal of approval’. As Carter et al. put it, public trust and a social licence can only be gained when ‘reasonable citizens can […] recognise [an activity, such as data sharing] as defensible on the grounds that it reflects common social values and goals’ [8]. Yet these values and goals are put into question by the new partnerships between the NHS and commercial bodies. The question arises then as to if, and if so how, the NHS can maintain its solidaristic character and hence, public trust regarding data sharing, when more and more commercial interests enter the public healthcare sector.

### Maintaining solidarity and public trust in a cash-strapped NHS

It appears that in order for people to maintain their trust in the NHS and agree to their data to be used for purposes beyond direct health benefit including (academic and commercial) research and economic benefit, the NHS needs to uphold its solidaristic character. As outlined above, this seems increasingly challenging in a cash-strapped healthcare system, with more and more private companies entering the field, diluting its public character. Should partnerships with commercial entities be avoided altogether? Considering the potential of data-driven technologies to improve and personalise patient care and the fundamental role private companies play in developing such technologies, this would seem, at least at this stage, unreasonable. Is it possible, then, for both the NHS and private digital companies that seek access to its data (e.g. Google, Amazon) to build trustworthy partnerships, and if so how? And, what would the social contract or licence^1^ that is the ‘common set of principles and values that bind together’ [7] the NHS and society look like in this new context?

As a minimum criterion, partnerships between the NHS and the private sector will need to be arranged in a way that preserve public trust and maintain the healthcare system’s solidaristic character [40]. This means that these partnerships will need to reflect in their structure, organisation and stated aims the basic principles of universality, equality and quality. The introduction of laws and regulations can go some way to convince people that their interests are protected. However, as Shapiro notes, trust often appears to have a paradoxically reverse relationship to laws and regulations; the more fidelity assurances one seeks through laws and regulations, the less trustworthy these assurances appear [41]. The pursuit of collective interests and the common good seems crucial to the establishment and preservation of trust [34] that needs to sit alongside strict regulations as offered by e.g. the EU General Data Protection Regulation [42] or the Big Data Task Force of European Medicines Agencies and the Heads of Medicines Agencies [43]. Yet, regulations, unless strictly and consistently enforced, can be neglected or overlooked by individual actors. As Carter et al. observe, trust in data use does not depend on, for example, the ‘formal architecture of research regulation, or on rational assessment of the detail of information sheets or other documents aimed at gaining ‘informed consent’’ [8]. Rather, trust in the use of their data by commercial companies developing new tools relies ‘heavily on its status as a socially valuable enterprise conducted in the service of the public good’.

However, a selfless commitment to the public good might appear to be at odds with the interests of profit-oriented private companies partnering with the NHS. Is it, therefore, reasonable to suggest that these partnerships could be founded on public benefit rather than shareholders’ profit? Governance arrangements that place the common good at the core of the partnership could offer an acceptable approach to this. When designing and negotiating partnerships and collaborations with private and commercial companies, NHS officials and the government should build these on norms of solidarity and public benefit and also take note of the various suggestions included in the published reports on public views and expectations regarding the use of patient data, and also where its ‘red lines’ lie. This would provide them not only with a useful insight but also a roadmap to draft arrangements that foster the social contract or licence with the public, convince them of their commitment to solidaristic principles and maintain trust. Following these recommendations, the NHS could: (1) require preferential access to goods and services developed using its data [6]; (2) require that data are used only to improve health and healthcare and not to serve the interests of private insurance companies or other commercial interests [18, 44]; (3) conflicts of interests partners might have are publicly declared, managed and resolved; (4) use a monitored data-visiting model of access similar to the one developed by Genomics England, rather than a data-sharing model where the data leaves the NHS custodianship. The NHS national data opt-out policy argues that giving patients more say and active control over their own data could preserve public trust in the healthcare system [45]. Notwithstanding the importance of allowing individuals the option to express their own preferences and values that an opt-out system presents, placing too much emphasis on the responsibility of the individual to take active control over their data seems at odds with the basic characteristics of solidarity and public trust. Firstly, requiring patients to take individual responsibility for the use of their data implies a relationship of mistrust rather than trust. Requiring individual to protect their own interests could be perceived as the system’s inability or reluctance to fulfil its role of acting in the public’s best interest. Secondly, by relying on people to take control of their own data, the system might end up undermining the public good it is tasked with serving. Rather, it becomes a manager of individual preferences, thus losing its solidaristic characteristics of universality and equality. Thirdly, by inviting people to think and act as individuals protecting their own interests, the feelings of mutuality and commonality, foundational for solidarity, get weakened and the bond between society members might be lost [25].

If the NHS is to remain the most supported and trusted institution in the UK, using data-driven technologies to provide more accurate prediction and prevention of disease, improve health outcomes and more efficient use of resources, it will need to retain its foundational principle of serving the public good, and promote the beliefs of mutuality and commonality in society. Only if it can prove its continuous solidaristic character, the NHS will succeed in maintaining public trust and in negotiating a new social contract or licence.

## Conclusion

People trusts solidaristic institutions that openly preserve collective interests, and promote collective wellbeing. In the UK, the solidaristic character of the NHS makes it one of the most trusted public institutions. In recent years, however, public trust has been challenged due to private companies collaborating with the NHS and been given access to its patient data. Despite the potential benefits of data-driven healthcare (e.g. more accurate prediction and prevention of disease, improved health outcomes and more efficient use of resources), partnerships with profit-oriented companies do not necessarily chime with the NHS’s foundational principles of universality and equity. Consequently, trust in the NHS is put to the test.

In order to, nevertheless, maintain public trust without forgoing the potential benefits of data-driven healthcare technologies, the NHS needs to put in place solidarity grounded partnerships with companies seeking to access patient data. Such partnerships would need to serve collective interests through, for example, securing preferential access to goods and services, providing health benefits, and monitoring data access. This approach will help establish a social contract or licence that responds to expectations and principles of a solidaristic healthcare system. As the current health minister rightly observed, unless the NHS maintains public trust, the vision of a data-driven healthcare will not be realisable.

Although our analysis focuses on the UK context, it contributes to the wider bioethics discussion regarding the governance of data sharing between public healthcare systems and commercial companies.

## Data Availability

Not applicable. This is a literature-based manuscript. No data was collected.

## Abbreviation

NHS: National Health Service

## Footnotes

1 Both terms describe a set of values and norms defining responsibilities and rights that are im-/explicitly ‘agreed upon’ in a society. Whereas the social licence focuses more on procedural agreements, the term social contract refers to the ‘broader context of social arrangements, practices and institutions’ (7.Lucassen A, Montgomery J, Parker M. Chapter 16. Ethics and the social contract for genomics in the NHS. Annual Report of the Chief Medical Officer 2016: Department of Health; 2017.).
